# HIV Promoters Isolated from Brain and Peripheral Tissue of Virally Suppressed PWH Are Phylogenetically and Functionally Similar

**DOI:** 10.3390/ijms27073185

**Published:** 2026-03-31

**Authors:** Janna Jamal Eddine, Emily K. Chalmers, Jingling Zhou, Sarah J. Byrnes, Trisha A. Jenkins, Narin Osman, Anna C. Hearps, Michael Roche, Thomas A. Angelovich, Melissa J. Churchill

**Affiliations:** 1 ATRACT Leading Research Centre, School of Health and Biomedical Sciences, RMIT University, Melbourne 3000, Australia; janna.jamal.eddine@rmit.edu.au (J.J.E.); sarah.byrnes@rmit.edu.au (S.J.B.); thomas.angelovich@rmit.edu.au (T.A.A.); 2 Life Sciences Discipline, Burnet Institute, Melbourne 3004, Australia; 3 Department of Infectious Diseases, The University of Melbourne, Peter Doherty Institute for Infection and Immunity, Melbourne 3000, Australia; 4 Department of Infectious Diseases, Monash University, Melbourne 3800, Australia; 5 Departments of Microbiology and Medicine, Monash University, Melbourne 3800, Australia

**Keywords:** HIV, brain, reservoir, LTR, compartmentalization, sequencing, transcription factor binding sites

## Abstract

Despite viral suppression with antiretroviral therapy (ART), reservoirs of Human Immunodeficiency Virus (HIV) persist in anatomical compartments throughout the body, including the brain. We have previously demonstrated that the HIV long terminal repeats (LTRs) isolated from the brains of non-virally suppressed people with HIV (PWH) are phylogenetically and functionally distinct from those isolated from matched peripheral tissue. While intact, transcriptionally competent HIV genomes persist within the brains of virally suppressed PWH, whether HIV LTRs are intact, functional, and compartmentalized relative to the periphery, as in non-virally suppressed PWH, remains unclear. HIV LTRs were extracted from frontal cortex post-mortem brain and matched peripheral tissues of virally suppressed PWH (*n* = 5). Following single-genome amplification, sequences were phylogenetically analyzed and transcriptional activity was assessed. In contrast to non-virally suppressed PWH, LTR sequences failed to compartmentalize between the brain and peripheral compartments. Identical LTR sequences were observed across brain and peripheral tissues in 2/5 PWH. While the LTRs remain transcriptionally active, mutations, insertions and deletions predicted to reduce transcription factor binding affinity at key binding sites, including C/EBP, NF-κB, and Sp1 sites, were observed and found to result in reduced basal transcriptional activity. The role of these mutations in latency and viral persistence remains unclear.

## 1. Introduction

The persistence of human immunodeficiency virus (HIV) in cellular and tissue reservoirs is a major barrier to cure and a potential contributor to comorbid diseases that affect people with HIV despite viral suppression with antiretroviral therapy (ART). Numerous studies have identified latently infected CD4+ T cells and tissue resident cells, including macrophages, across various anatomical compartments, including the brain. These reservoirs can provide a source of ongoing viral transcription, translation and, in certain circumstances, viral rebound during treatment interruption. Ongoing activation of HIV proviruses in reservoirs may also contribute to cellular activation and pathology leading to comorbid disease, including cancer, liver disease, cardiovascular disease, and, importantly, neurocognitive impairment [1,2,3,4], highlighting the need to define the transcriptional activity of HIV reservoirs in the body, including the brain.

HIV enters the central nervous system (CNS) during acute infection through the migration of infected T cells and macrophages across the blood–brain barrier. This leads to infection of cells within the cerebrospinal fluid (CSF) and brain parenchyma, including macrophages and microglia, which may contribute to neurocognitive decline [5,6]. Importantly, we have previously demonstrated that a HIV reservoir persists in the brain during suppressive ART at similar levels present in non-virally suppressed people with HIV (PWH) [7,8], supporting a stable reservoir of HIV despite ART. Furthermore, multiple studies, including our own, have demonstrated the presence of intact, potentially replication-competent HIV provirus in the brains of virally suppressed PWH [7,8,9]. In addition, we recently demonstrated that HIV RNA transcripts persist in the frontal cortex of virally suppressed PWH, suggesting ongoing transcription despite undetectable plasma viral loads [10]. Viral persistence and activation may contribute to cellular activation, neuroinflammation and poorer cognitive outcomes [11,12,13,14]. However, the mechanisms governing HIV persistence and the generation of HIV RNA transcripts in the brains of virally suppressed PWH are unclear.

Transcription of HIV is regulated by long terminal repeats (LTRs) that flank the integrated HIV genome. These LTRs contain transcription factor binding sites (TFBSs) for cellular factors that initiate and regulate viral transcription [15,16,17]. The core/enhancer region of an LTR contains binding motifs for NF-κB, Sp1 and C/EBP cellular factors, while the R region contains the TAR element that is responsible for the recruitment of the viral transactivator Tat [15,18,19]. Binding of cellular proteins to TFBSs initiates transcription, regulates chromatin accessibility, and determines the balance between latency and reactivation [20,21,22]. Thus, the analysis of LTR sequences provides critical insight into the transcriptional potential of the viral reservoir.

Previous work from our laboratory and others has shown that HIV LTR sequences derived from parenchymal brain tissue of non-virally suppressed PWH are genetically and functionally distinct from those from matched peripheral tissues [23,24,25,26,27,28]. Specifically, we demonstrated that point mutations both within and flanking Sp1 sites of brain-derived LTRs reduced Sp1 binding affinity and diminished transcriptional activity relative to peripheral LTRs lacking these changes [24]. We and others have also demonstrated that HIV *env* sequences phylogenetically cluster independently, demonstrating phylogenetic compartmentalization between brain and peripheral compartments of non-virally suppressed PWH [27,29,30]. This distinction in sequences isolated from the brain and peripheral compartments likely indicates CNS-specific viral evolution. In contrast, recent studies have demonstrated that *env* sequences isolated from virally suppressed PWH fail to compartmentalize between CNS and non-CNS tissues [31,32,33,34,35], likely reflecting a lack of ongoing replication within compartments in the presence of ART. Specifically, a study analyzing full-length *envs* isolated from brain parenchyma or peripheral tissues demonstrated compartmentalization of sequences in only 2/10 ART-treated PWH [35]. Whether this lack of compartmentalization also applies to HIV LTRs, and importantly, whether they retain distinct transcriptional properties under viral suppression, has not been established.

In this study, HIV LTRs isolated from the brain and matched peripheral tissues of virally suppressed PWH were analyzed for intactness, evidence of phylogenetic compartmentalization, their ability to bind transcription factors, and their capacity to drive transcription in vitro.

## 2. Results

### 2.1. HIV LTR Sequences from Virally Suppressed PWH Do Not Phylogenetically Compartmentalize Between Brain and Peripheral Compartments

To determine the genetic relationship between HIV LTRs derived from CNS and non-CNS compartments during viral suppression, LTR sequences were examined from a cohort of five PWH who were virally suppressed at the time of death. Fresh frozen frontal cortex brain tissue and matched peripheral non-brain tissue (either spleen, lymph node or gastrointestinal tract, where available) were obtained from the National NeuroHIV tissue consortium (NNTC). The time between death and autopsy (post-mortem interval) was a median of 7.5 h. Participants were virally suppressed with ART (defined as undetectable viral load) for a median of 3.4 years, had a median CD4+ T cell count of 328 cells/mm^3^ and were on antiretroviral regimens with medium ART CNS penetrance efficacy at the time of death (Table S1).

**Figure 1:**
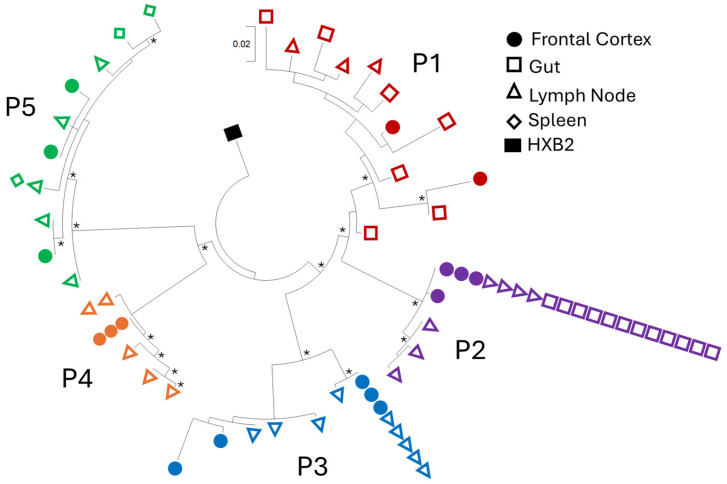
LTR sequences isolated from frontal cortex brain tissue do not compartmentalize from peripheral LTR sequences in virally suppressed PWH. HIV LTRs were isolated from frontal cortex brain tissue (closed circle) and peripheral tissue, including lymph node (open triangle), spleen (open diamond) or gastrointestinal tract (open square). Maximum likelihood tree anchored to the HXB2 laboratory isolate (closed rectangle) was performed with 1000 bootstrap replicates generated; bootstrap values over 70 are indicated with an asterisk (*). Each color represents a single PWH: P1 (red), P2 (purple), P3 (blue), P4 (orange) and P5 (green).

A total of 66 HIV LTR sequences were obtained from the brain and matched peripheral tissues of five virally suppressed PWH, including 41 unique sequences (Table S2). As expected, LTR sequences clustered by individual PWH (Figure 1); however, no evidence of phylogenetic compartmentalization between isolates from frontal cortex and peripheral tissues was observed in virally suppressed PWH, contrasting with previous findings in non-virally suppressed PWH [23].

LTR sequences did not compartmentalize between tissue compartments for all PWH. In P1, sequences from the gut, lymph node and brain clustered together, with no clear compartmentalization of brain sequences, as was previously observed in non-virally suppressed cohorts [23,24]. Identical sequences were also detected across compartments in a subset of PWH. In P2, three brain-derived isolates, four lymph node isolates, and twelve gastrointestinal isolates exhibited 100% sequence homology. Similarly, in P3, three brain isolates and five lymph node isolates were identical. In P5, identical LTRs were observed between lymph node and spleen isolates, although no identical brain sequences were observed.

These findings indicate that in virally suppressed PWH, LTR sequences isolated from the brain do not phylogenetically compartmentalize from those isolated from peripheral tissues, with some evidence of identical LTRs present across multiple tissue sites.

### 2.2. Mutations in Transcription Factor Binding Sites Are Observed to Be Heterogeneous Across Brain and Peripheral LTRs

**Figure 2:**
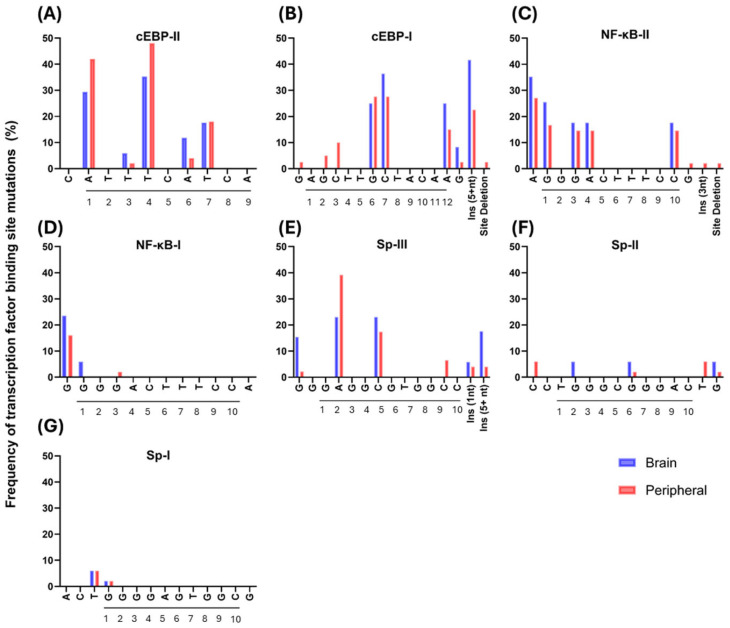
Frequency of mutations within key transcription factor binding sites of HIV LTRs isolated from brain and peripheral tissues of virally suppressed PWH. HIV LTRs (*n* = 66) were isolated from frontal cortex (blue) and peripheral tissues (red; lymph node, gastrointestinal tract, and spleen). Multiple sequence alignments were generated, and the frequency of point mutations relative to the HXB2 reference was calculated using AnalyzeAlign (https://www.hiv.lanl.gov/content/sequence/ANALYZEALIGN/analyze_align.html, accessed on 1 October 2025). Core binding sites are underlined, and positions within each site are numbered. Sites analyzed included (**A**) C/EBP-II (HXB2 position −173 to −165), (**B**) C/EBP-I (−117 to −104), (**C**) NF-κB-II (−105 to −94), (**D**) NF-κB-I (−91 to −80), (**E**) Sp-III (−79 to −66), (**F**) Sp-II (−68 to −55), and (**G**) Sp-I (−58 to −45).

To assess the distribution of specific mutations within key TFBSs, which could be responsible for HIV latency/altered transcriptional activity, we analyzed mutation frequencies within the consensus sites and adjacent nucleotides of key motifs, including C/EBP-II, C/EBP-I, NF-κB-II, NF-κB-I, Sp-III, Sp-II, and Sp-I of brain and peripheral LTRs (Figure 2). Sequences with insertions and deletions were removed from the analysis and reported independently. Sp-I and Sp-II sites were highly conserved, but Sp-III, which has been previously reported to play the most significant role in HIV transcription [24], had multiple mutations at core positions 2 and 5 and within the 5′ adjacent site. These occurred in both brain (15–23%) and peripheral (2–39%) LTRs. An additional Sp site was present in 17% of brain-derived sequences and 4% of peripheral sequences; however, these were all derived from a single participant (P4).

C/EBP-I and C/EBP-II sites were the least conserved sites examined. C/EBP-II core positions 1 and 4 were highly variable for brain (24–35%) and peripheral isolates (42–48%). Similarly, C/EBP-I was highly variable in positions 6, 7 and 12 for brain (25–36%) and peripheral isolates (15–28%). In addition, insertions of at least 5 nt were observed in 42% of brain isolates and 23% of peripheral isolates.

### 2.3. Observed Sequence Variation in HIV LTRs from Virally Suppressed PWH Is Predicted to Reduce Transcription Factor Binding

Find Individual Motif Occurrences (FIMO) can be used to assess the potential functional impact of sequence variation within transcription factor binding sites of known consensus binding motifs [36]. FIMO was applied to regions encompassing key TFBSs (Sp, NF-κB, and C/EBP) in sequences isolated from virally suppressed PWH. Unique sequence variants of Sp, NF-κB and C/EBP sites relative to HXB2 were identified and analyzed using FIMO to generate predicted binding scores, where higher scores indicate stronger predicted binding affinity.

#### 2.3.1. Sp1 Binding Variants

**Figure 3:**
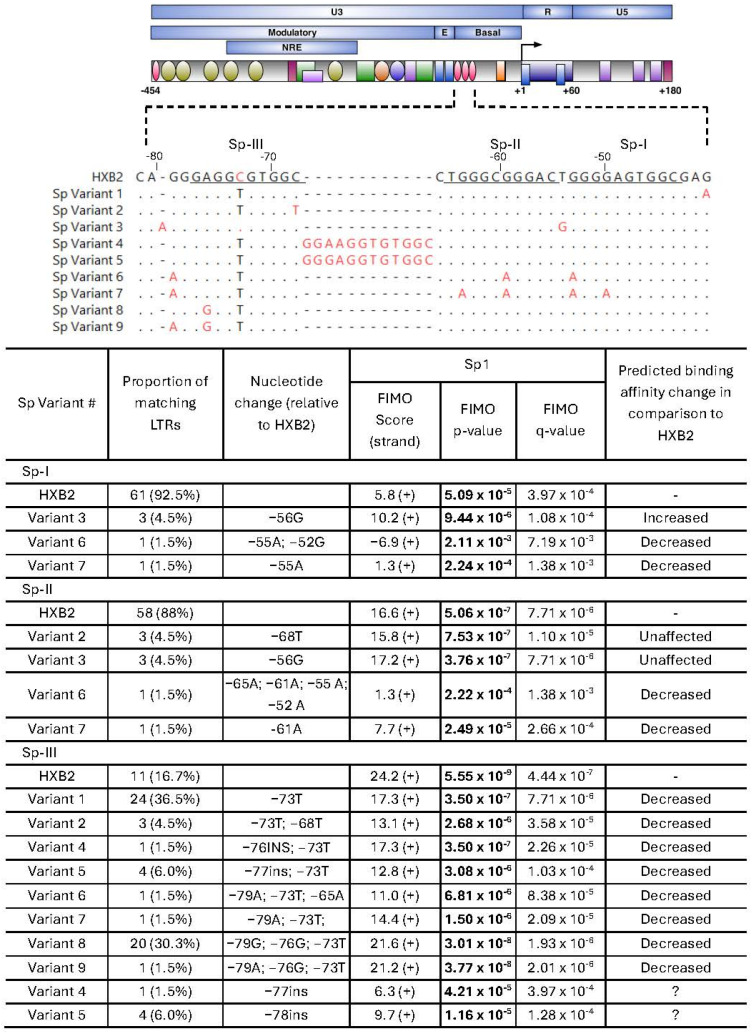
Unique Sp sequence variants isolated from virally suppressed PWH. (**Top**) Schematic of the HIV LTR showing transcription factor binding sites and a multiple sequence alignment of unique Sp site variants relative to HXB2, with nonmatching nucleotide residues indicated in red. Core Sp transcription factor binding sites are underlined. (**Bottom**) Predictive binding affinity of unique Sp site variants using Find Individual Motif Occurrences (FIMO). Positive (+) and negative (−) DNA strands were searched, with the strand yielding the higher score reported. Higher FIMO scores indicate stronger predicted binding affinity. Statistically significant values (*p* < 0.05) are shown in bold; *q*-values represent the false discovery rate-adjusted *p*-value. Abbreviations: ins: insertion.

Sequence variation within the Sp motifs was analyzed to determine potential effects on predicted motif recognition. A total of nine Sp site variants were identified across brain and peripheral LTRs, comprising three unique Sp-I variants, three unique Sp-II variants and eight unique Sp-III variants (Figure 3). The binding of the Sp1 protein to each of these site variants was assessed. Among the HXB2 reference sites, Sp-I had the lowest predicted Sp1 binding score (FIMO score: 5.8; *p* = 5.09 × 10^−5^; q = 3.97 × 10^−4^), followed by Sp-II (16.6; *p* = 5.06 × 10^−7^; q = 7.71 × 10^−6^) and Sp-III (24.2; *p* = 5.55 × 10^−9^; q = 4.44 × 10^−7^).

Most Sp-I sequences observed (92.5%) were identical to HXB2. Variants 6 and 7 had a markedly reduced predicted Sp1 binding score (−6.9; *p* = 2.11 × 10^−3^, q = 7.19 × 10^−3^), suggesting Sp1 binding was unlikely. Variant 7 also showed substantially reduced predicted binding when compared to HXB2 (1.3; *p* = 2.24 × 10^−4^; q = 1.38 × 10^−3^). In contrast, variant 3, which contained a T → G mutation in between core Sp-I and Sp-II sites (HXB2 position −56), displayed increased predicted Sp1 binding relative to HXB2 for both Sp-I (10.2; *p* = 9.44 × 10^−6^; q = 1.08 × 10^−4^) and Sp-II sites (17.2; *p* = 3.76 × 10^−7^; q = 7.71 × 10^−6^). The remaining Sp-II variants (variants 2, 6 and 7) had decreased predicted binding affinity, with FIMO scores as low as 1.3 (variant 6).

A common C → T mutation found in Sp-III (HXB2 position −74) was identified in eight variants, present in 55/66 LTR sequences. This mutation, when analyzed in isolation, had a FIMO score of 17.3 (*p* = 3.50 × 10^−7^; q = 7.71 × 10^−6^), indicating strong predicted binding, although lower than HXB2. Previous electrophoretic mobility shift assay data from our laboratory have shown that while direct Sp1:Sp-III binding remained strong in the presence of this mutation, overall Sp1 binding across all three sites was reduced [24], suggesting a cumulative decrease in binding affinity.

An additional Sp binding site was detected within variants 4 and 5, representing 7.5% of LTR sequences isolated. These novel sites were predicted to have weak Sp1 binding (variant 4: 6.3; *p* = 4.21 × 10^−5^, q = 3.97 × 10^−4^; variant 5: 9.7; *p* = 1.16 × 10^−5^, q = 1.28 × 10^−4^); however, the effect on overall Sp1 binding and influence on chromatin structure remains unclear. Overall, although the majority of Sp1 sites were identical to HXB2, identified Sp1 site variants were predicted to reduce Sp1 binding relative to the HXB2 reference.

#### 2.3.2. NF-κB Binding Site Variants

**Figure 4:**
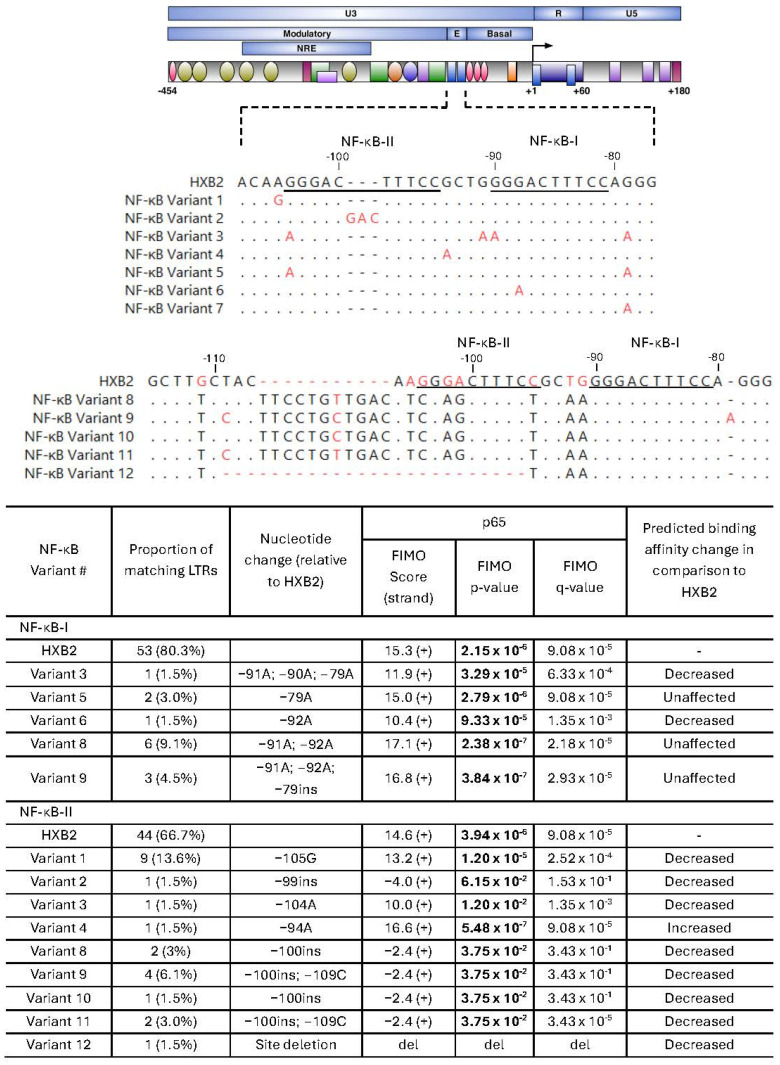
Unique NF-κB sequence variants isolated from virally suppressed PWH. (**Top**) Schematic of the HIV LTR showing transcription factor binding sites and a multiple sequence alignment of unique NF-κB site variants relative to HXB2, with nonmatching nucleotide residues indicated in red. Core NF-κB transcription factor binding sites are underlined. (**Bottom**) Predictive binding affinity of unique NF-κB site variants using Find Individual Motif Occurrences (FIMO). Positive (+) and negative (−) DNA strands were searched, with the strand yielding the higher score reported. Higher FIMO scores indicate stronger predicted binding affinity. Statistically significant values (*p* < 0.05) are shown in bold. *q*-values represent the false discovery rate-adjusted *p*-value. Abbreviations: del: deletion, ins: insertion.

Variation in NF-κB was assessed to explore potential changes in predicted binding within this key regulatory motif. The NF-κB motif within the HIV LTR is essential for both basal and activated transcription [37,38]. A total of 12 distinct NF-κB motif variants were identified across all assessed LTRs (Figure 4). Most NF-κB-I sequences (80.3%) were identical to HXB2, with strong predicted binding to p65 (15.3; *p* = 2.15 × 10^−6^; q = 9.08 × 10^−5^). Five variants were identified, two with reduced predicted binding (variant 3: 11.9; *p* = 3.29 × 10^−5^, q = 6.33 × 10^−4^ and variant 6: 10.4; *p* = 9.33 × 10^−5^, q = 1.35 × 10^−3^), two with enhanced predicted binding (variant 8: 17.1; *p* = 2.38 × 10^−7^; q = 2.18 × 10^−5^ and variant 9: 16.8; *p* = 3.84 × 10^−7^; q = 2.93 × 10^−5^) and one with no notable change (variant 5: 15.0; *p* = 2.79 × 10^−6^; q = 9.08 × 10^−5^).

Greater variability was observed in NF-κB-II, with only 66.7% of sequences identical to HXB2, which had strong predicted binding (14.6; *p* = 3.94 × 10^−6^; q = 9.08 × 10^−5^). Five variants had a predicted loss of binding (variants 2, 8, 9, 10, and 11; FIMO scores < 0), and one variant had a deleted NF-κB-II site (variant 12). Variants 1 and 3 showed reduced binding (variant 1: 13.2, *p* = 1.20 × 10^−5^, q = 2.52 × 10^−4^; variant 3: 10.0, *p* = 1.20 × 10^−4^, q = 1.35 × 10^−3^) compared to HXB2. While variant 4 demonstrated an increase in predicted binding (16.6, *p* = 5.48 × 10^−7^, q = 9.08 × 10^−5^), this was only identified in 1.5% of LTRs. Overall, the majority of NF-κB site variants were predicted to reduce NF-κB binding relative to the HXB2 reference.

#### 2.3.3. C/EBP Binding Site Variants

**Figure 5:**
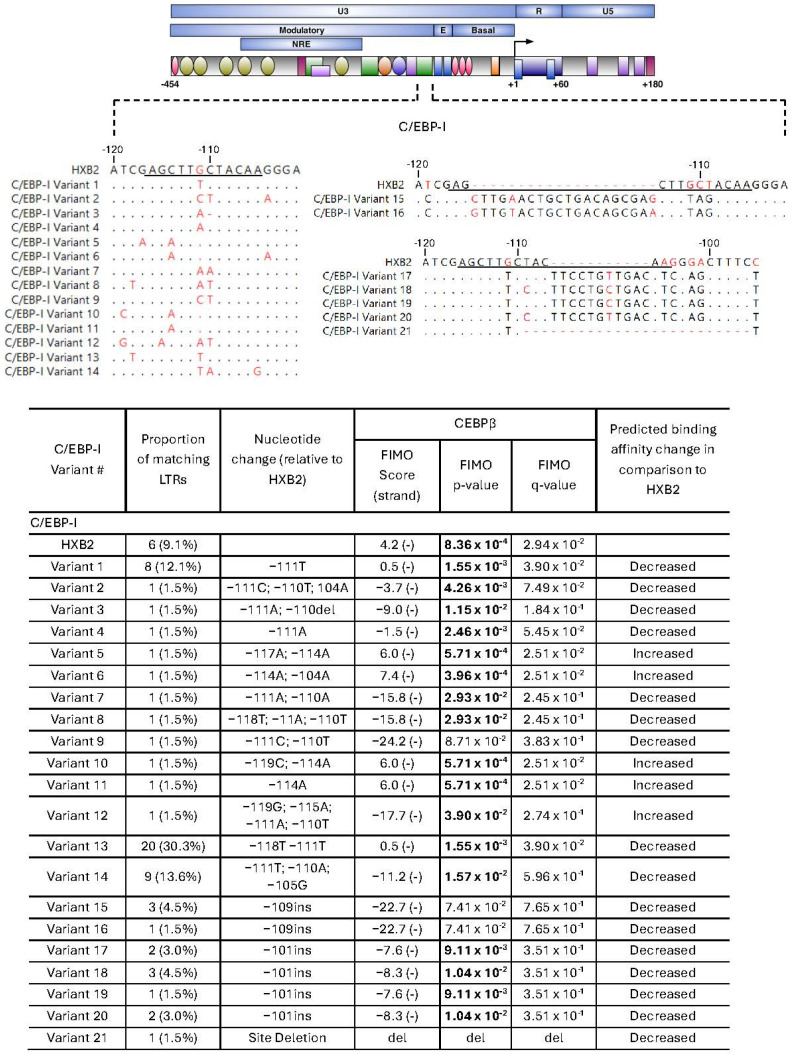
Unique C/EBP-I sequence variants isolated from virally suppressed PWH. (**Top**) Schematic of the HIV LTR showing transcription factor binding sites and a multiple sequence alignment of unique C/EBP-I site variants relative to HXB2, with nonmatching nucleotide residues indicated in red. Core C/EBP-I transcription factor binding sites are underlined. (**Bottom**) Predictive binding affinity of unique C/EBP-I site variants using Find Individual Motif Occurrences (FIMO). Positive (+) and negative (−) DNA strands were searched, with the strand yielding the higher score reported. Higher FIMO scores indicate stronger predicted binding affinity. Statistically significant values (*p* < 0.05) are shown in bold. q values represent the false discovery rate-adjusted *p*-value. Abbreviations: del: deletion; ins: insertion.

**Figure 6:**
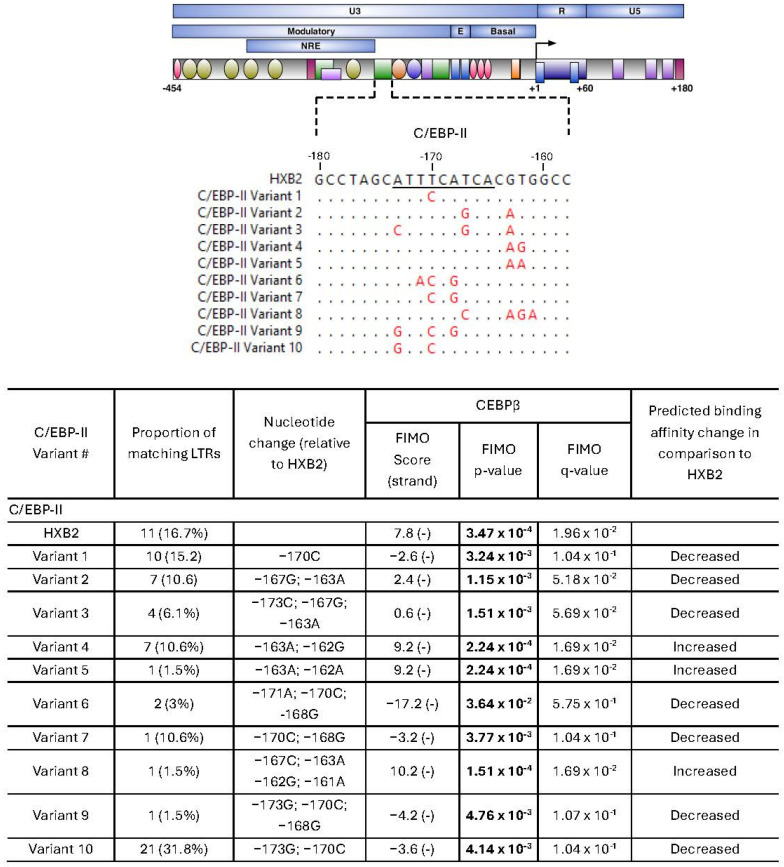
Unique C/EBP-II sequence variants isolated from virally suppressed PWH. (**Top**) Schematic of the HIV LTR showing transcription factor binding sites and a multiple sequence alignment of unique C/EBP-II site variants relative to HXB2, with nonmatching nucleotide residues indicated in red. Core C/EBP-II transcription factor binding sites are underlined. (**Bottom**) Predictive binding affinity of unique C/EBP-II site variants using Find Individual Motif Occurrences (FIMO). Positive (+) and negative (−) DNA strands were searched, with the strand yielding the higher score reported. Higher FIMO scores indicate stronger predicted binding affinity. Statistically significant values (*p* < 0.05) are shown in bold. *q*-values represent the false discovery rate-adjusted *p*-value.

C/EBPβ plays an important role in regulating both basal and Tat-activated HIV transcription, particularly in monocyte/macrophage lineage cells [25,39,40,41,42,43,44]. A total of 21 distinct C/EBP-I variants (Figure 5) and 10 C/EBP-II variants were identified (Figure 6).

C/EBP-I sites were highly variable, with only 9.1% of variants identical to HXB2. The HXB2 site had weak predicted C/EBPβ binding affinity (4.2; *p* = 8.36 × 10^−4^, q = 2.94 × 10^−2^). The most common configuration, variant 13, further reduced predicted binding (0.5, *p* = 1.55 × 10^−3^, q = 3.90 × 10^−2^) and was present in 30.3% of sequences. Only four variants increased predicted C/EBPβ binding, including variant 5 (6.0, *p* = 5.71 × 10^−4^, q = 2.51 × 10^−2^), variant 6 (7.4; *p* = 3.96 × 10^−4^; q = 2.51 × 10^−2^) variant 10 (6.0; *p* = 5.71 × 10^−4^; q = 2.51 × 10^−2^) and variant 11 (6.0; *p* = 5.71 × 10^−4^; q = 2.51 × 10^−2^). The remaining variants had either very weak predicted or no binding (FIMO score < 0.5), including variant 21, which contained a site deletion.

The C/EBP-II site was also highly variable, with 16.7% of isolates identical to HXB2. C/EBP-II had stronger predicted binding than C/EBP-I in HXB2 (7.8; *p* = 3.47 × 10^−4^; q = 1.96 × 10^−2^). Variant 10 was the most common configuration, representing 31.8% of sequences, but was predicted not to bind C/EBPβ (−3.6; *p* = 4.14 × 10^−3^, q = 1.04 × 10^−1^). Variants 4, 5 and 8 had increased predicted binding (variant 4 (9.2; *p* = 2.24 × 10^−4^; q = 1.69 × 10^−2^), variant 5 (9.2; *p* = 2.24 × 10^−4^; q = 1.69 × 10^−2^) and variant 8 (10.2; *p* = 1.51 × 10^−4^; q = 1.69 × 10^−2^)). The remaining variants had weak predicted binding (variants 2 and 3; FIMO score < 2.4) or no binding (variants 1, 6, 7, 9, and 10; FIMO score < 0). Overall, the majority of C/EBP site variants were predicted to reduce or abolish C/EBPβ binding relative to the HXB2 reference.

Overall, sequence variation was observed in key transcription factor binding sites, including Sp1, NF-κB and C/EBP, with most variants either maintaining or reducing predictive motif binding. These variants were not limited to a particular anatomical compartment and may result in a reduced transcriptional activity.

### 2.4. No Tissue-Specific Transcriptional Activity Was Observed in LTRs Isolated from Brain and Peripheral Tissues of Virally Suppressed PWH

**Figure 7:**
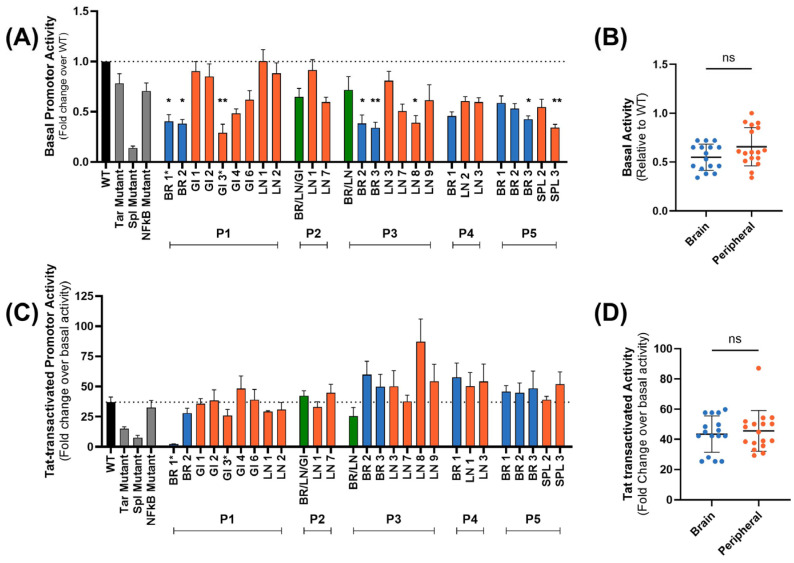
Basal and Tat-activated transcriptional activity of brain- and peripheral-derived LTRs from virally suppressed PWH. (**A**,**B**) Basal and (**C**,**D**) Tat-activated transcriptional activity of HIV LTR isolates from frontal cortex brain (BR; blue), lymph node (LN; red), gastrointestinal tract (GI; red) or spleen (SPL; red) tissue in the microglial cell line HMC3s. Transcriptional activity of HIV LTRs isolated from multiple tissue compartments are shown in green. Basal promoter activity is expressed relative to wild-type (HXB2 LTR) activity. Tat-activated promoter activity is expressed as a fold change over basal activity. Wild-type LTR is shown in black; TAR mutated, Sp1-II mutated and NF-κB-II mutated controls are shown in gray. Error bars represent the mean with SEM across technical replicates. ANOVA Kruskal–Wallis with Dunn’s multiple comparison test was used to compare each individual participant’s LTR to wild-type activity. Brain and peripheral LTR activities were compared using a non-parametric Mann–Whitney U test. A *p*-value < 0.05 was considered statistically significant (ns: not significant, * *p* < 0.05, ** *p* < 0.01).

Transcriptional activity from LTRs isolated from brain and peripheral tissues from virally suppressed PWH was analyzed in brain cells ex vivo. Basal transcriptional activity was heterogeneous and independent of the anatomical compartment of origin (Figure 7A), and no significant difference in activity was observed (Figure 7B; *p* = 0.1754). Tat-mediated activation of LTRs was also heterogeneous (Figure 7C), with levels comparable to the consensus HXB2 (Figure 7C; *p* > 0.05), and no significant difference in activity was observed between brain and peripheral LTRs (Figure 7D; *p* = 0.9220). Together with the sequence variation analyses, these functional data suggest that in the context of ART, HIV LTRs remain capable of viral transcription. Furthermore, HIV LTRs isolated from brain and peripheral tissues retain similar transcriptional activity, which contrasts with findings in non-virally suppressed PWH [24].

## 3. Discussion

Despite the effectiveness of ART in suppressing systemic viremia, ART alone does not eradicate HIV infection. Virally suppressed PWH remain at increased risk of comorbidities, including neuropathology, which is attributed in part to the persistence of HIV within long-lived cellular reservoirs [3,4,45]. The mechanisms that facilitate HIV persistence and latency remain incompletely understood but are thought to involve transcriptional regulatory adaptations within the viral LTR, which governs viral gene expression. Understanding the genetic and functional properties of HIV LTRs within tissue reservoirs, particularly the CNS, is critical to defining mechanisms of viral persistence during viral suppression.

Prior to the widespread use of ART, in viremic individuals, compartmentalization of viral sequences between the CNS and peripheral tissues was a well-established feature of HIV infection, particularly for *env* and LTR regions [23,29,46]. This compartmentalization could reflect local viral replication and independent evolution within CNS cellular reservoirs, such as perivascular macrophages and microglia, resulting in genetically and functionally distinct viral populations. In the present study, we investigated whether such compartmentalization is maintained in virally suppressed PWH receiving long-term ART and whether these LTRs were intact and capable of driving viral transcription. A total of 66 HIV LTR sequences were isolated from multiple anatomical compartments, including frontal cortex, lymph node, gastrointestinal tract, and spleen tissues from five virally suppressed individuals. Phylogenetic analysis revealed clustering by individual rather than anatomical compartment, with brain-derived and peripheral tissue-derived sequences intermixed. Notably, identical LTR sequences were identified across CNS and peripheral tissues in some individuals, an observation not reported in untreated cohorts.

These findings contrast with our previous observations and those of others, demonstrating clear compartmentalization of HIV LTR and *env* sequences between CNS and peripheral tissues in non-suppressed PWH [23,24,27,29]. The absence of compartmentalization under viral suppression likely reflects fundamental differences in the biology of persisting viruses compared with those present during active viremia. During untreated infection, ongoing viral replication within CNS-resident macrophages, microglia, and infiltrating T cells likely drives genetic diversification and local adaptation, resulting in distinct compartment-specific viral populations. In contrast, the intermixed phylogenetic relationships and presence of identical sequences across compartments observed here suggest that viruses persisting during viral suppression represent archival genomes that were seeded during earlier stages of infection and have remained genetically stable in the absence of ongoing replication. These findings are consistent with previous studies demonstrating identical or clonal pro-viral sequences across brain and peripheral tissues using near full-length pro-viral and *env* sequencing, supporting the concept that long-lived, archival genomes persist within distributed tissue reservoirs, including the CNS [33,35,47].

Sequence analysis revealed mutations across key TFBSs, including Sp1, NF-κB, and C/EBP motifs, which play central roles in regulating HIV transcription. Most observed mutations were predicted to either maintain or reduce transcription factor binding affinity. Variation within C/EBP binding sites was particularly notable, including mutations in C/EBP-I and C/EBP-II that reduced or abolished predicted binding. These sites are known to regulate HIV transcription in monocytes and macrophages, key reservoir cell types within the brain [25,40,42,43]. Previous functional studies have demonstrated that disruption of C/EBP binding sites can significantly impair basal transcriptional activity, highlighting their importance in promoter regulation [25,48]. Similarly, a recurrent C → T mutation at the Sp-III site was identified in the majority of sequences. Although motif prediction analysis suggested preserved binding, prior DNA–protein interaction studies from our laboratory demonstrated that this mutation impairs cumulative Sp1 recruitment across all three Sp sites, suggesting that even subtle nucleotide changes may have meaningful functional consequences for promoter activity [24].

Despite these sequence variations, LTRs derived from both brain and peripheral tissues remained transcriptionally competent, supporting both basal and Tat-activated transcription in vitro. Importantly, no significant functional differences were observed between brain-derived and peripheral-derived LTRs, indicating that transcriptional competence is preserved across anatomical reservoirs during viral suppression. This contrasts with previous observations in untreated PWH, where mutations within TFBSs, particularly in brain-derived LTRs, were associated with impaired transcriptional activity and compartment-specific functional differences [23,24]. The preservation of similar transcriptional activity across compartments under ART further supports the notion that persisting viral genomes represent a common archival population rather than independently evolving compartmentalized variants. Future studies assessing the transcriptional activity of brain and peripheral LTR variants in a variety of CNS and peripheral cell lines may further support this model.

Insertions and deletions within the LTR were also observed and were associated with reduced basal transcriptional activity. These structural alterations likely influence chromatin organization, including the positioning of nucleosomes relative to critical regulatory elements such as the nucleosome-free region between nuc-0 and nuc-1, which governs transcriptional accessibility [49]. Notably, the reduction in basal transcription associated with these alterations was largely overcome during Tat-mediated activation, suggesting that while basal promoter activity may be constrained, the capacity for reactivation remains intact. This functional profile is consistent with viral latency, wherein basal transcription is suppressed but inducible under appropriate activating conditions.

Collectively, these findings suggest that HIV LTRs persisting during long-term viral suppression retain functional transcriptional capacity but exhibit adaptations that reduce basal transcriptional activity. Mutations affecting key TFBSs, including Sp1, NF-κB, and C/EBP, were observed across both brain and peripheral tissues and were predicted to impair transcription factor binding. Compared with wild-type reference sequences and LTRs derived from non-suppressed individuals, these variants appear to exhibit reduced intrinsic transcriptional strength. Such adaptations likely confer a selective advantage by promoting viral latency, reducing spontaneous viral gene expression, and facilitating immune evasion while preserving the capacity for reactivation when host or cellular conditions permit.

Importantly, the lack of phylogenetic and functional compartmentalization between CNS and peripheral tissues indicates that brain reservoirs under viral suppression do not represent genetically or transcriptionally distinct viral populations. Instead, CNS reservoirs appear to harbor archival viral genomes that are phylogenetically and functionally similar to those in peripheral tissues [35]. This has important implications for HIV cure strategies, as it suggests that CNS reservoirs are not uniquely adapted at the LTR level but rather represent part of a distributed, transcriptionally competent latent reservoir. These findings also pose an increased challenge for cure studies, given the unique microenvironment of the brain parenchyma. Therapeutic strategies aimed at reversing viral latency or eliminating persistent reservoirs must therefore consider the shared transcriptional properties of HIV across both CNS and peripheral compartments. It is essential to consider whether therapeutics can cross the blood–brain barrier and achieve sufficient penetration into parenchymal tissue. Furthermore, potential off-target effects and the risk of inducing inflammatory responses must be carefully evaluated to avoid exacerbating neuropathogenesis and contributing to the development of neurocognitive disorders. In addition, future studies with increased sample size may be able to account for variables such as clade, population demographics and geographic regions, which may further inform treatment strategies.

In summary, HIV LTR sequences isolated from virally suppressed PWH demonstrate preserved transcriptional competence but exhibit mutations and structural alterations that reduce basal transcriptional activity. These LTRs are phylogenetically and functionally similar across brain and peripheral tissues, supporting the persistence of archival viral genomes across distributed anatomical reservoirs. These adaptations likely favor viral latency and long-term persistence under viral suppression, highlighting the importance of transcriptional regulation in maintaining HIV reservoirs and informing future cure strategies targeting latent infection.

## 4. Materials and Methods

### 4.1. Cohort

Human autopsy fresh frozen frontal cortex and matched peripheral tissues (either spleen, lymph node or gastrointestinal tract, where available) from virally suppressed PWH (*n* = 5; Table S2) were obtained with ethics approval and written approval from the National NeuroHIV Tissue Consortium, USA. Tissues were processed under ethics approval (RMIT University Human Research Ethics Committee #20843).

### 4.2. Single Genome Amplification of HIV LTR

HIV LTRs were isolated from the brain and peripheral tissues of virally suppressed PWH using single genome amplification. For efficient isolation of the 3’LTR, three rounds of nested PCR were optimized to isolate a final amplicon of ~672 bp (Table S3). Due to the nature of HIV reverse transcription, the 3’LTR was selected as it forms the 5’LTR in progeny virus and is therefore identical.

gDNA was extracted from 10 mg pieces of fresh frozen autopsy tissue using the AllPrep DNA/RNA/miRNA universal kit (Qiagen, Hilden, Germany; 80224) as per the manufacturer’s instructions. A 25 µL reaction was assembled using 12.5 µL Q5 High-Fidelity 2X Master Mix (NEB, Ipswich, MA, USA; M0492L), 1 µL of 10µM forward primer, 1 µL of 10 µM reverse primer, 1 µL of template DNA (up to 150 ng/reaction) and DNase/RNase-free distilled water (Invitrogen; 10977035). PCR thermocycling conditions were as follows: initial denaturation at 98 °C for 30 s, 35x cycles with 98 °C for 10 s, 60 °C (round 1) or 67 °C (round 2 and 3) for 30 s and 72 °C for 30 s, followed by a final extension at 72 °C for 2 min.

### 4.3. Phylogenetic Analysis of Viral Sequences

Amplicons were column-purified using the High Pure PCR Product Purification Kit (Roche, Basel, Switzerland; 11732668001), and nucleotide sequences were obtained via Sanger sequencing from the Australian Genome Research Facility. To ensure sequences were primary isolates and not laboratory contaminants, sequences were aligned against commonly used laboratory strains, including HXB2 and NL4-3, using CLC Main Workbench 24, and were further analyzed using NCBI BLAST (https://blast.ncbi.nlm.nih.gov/Blast.cgi, accessed on 1 August 2025). Individual participant sequence alignments were entered into the FindModel program (http://hiv.lanl.gov/content/sequence/findmodel/findmodel.html, accessed on 1 June 2025) to determine the model that best described the data. Duplicate sequences were removed to generate alignments of unique sequences only. Alignments were uploaded to MegaX, and maximum likelihood phylogenetic trees were constructed using the best identified model and standard settings with 1000 bootstrap replicates.

### 4.4. Individual Motif Analysis of Transcriptional Factor Binding Site

To identify mutations within key TFBSs relative to HXB2, individual alignments were generated for C/EBP-II, C/EBP-I, NF-κB-II, NF-κB-I, Sp-III, Sp-II, and Sp-I. Sequences containing insertions or deletions were excluded from the alignment and reported separately. Alignments were uploaded to AnalyseAlign (https://www.hiv.lanl.gov/content/sequence/ANALYZEALIGN/analyze_align.html, accessed on 1 October 2025) with the frequency threshold set to 100%.

To predict functional effects, human transcription factor position weight matrices were obtained from HOCOMOCO v11 (https://hocomoco11.autosome.org/, accessed on 1 October 2025; [50]) and scanned using FIMO (Find Individual Motif Occurrences; https://meme-suite.org/meme/doc/fimo.html, accessed on 1 October 2025; [36]). Canonical TFBSs were defined using HXB2, and unique participant TFBS sequences were compared accordingly. Scores were generated for both DNA strands, with the higher-scoring strand reported. Binding thresholds were defined as: score < 0 = non-binder, 1–5 = very weak, 5–10 = weak, 10–15 = moderate, and >15 = strong. LTR sequences were also trimmed to include only the TAR region, and secondary structure was predicted using CLC Main Workbench 24.

### 4.5. HIV LTR Transcriptional Activity Assays

To determine the transcriptional activity of participant-derived LTRs in vitro, LTRs were digested at the KpnI and HindIII sites and cloned into a pGL3-basic luciferase reporter vector already containing an HXB2-derived HIV LTR, as previously described [23,24]. HIV LTRs were transfected into the microglial cell line HMC3s (American Type Culture Collection; CRL-3304). A 96-well plate was seeded with 4000 cells/well and incubated overnight to achieve 40–60% confluency at the time of transfection. Cells were seeded and incubated in Eagle’s minimum essential medium (Sigma-Aldrich, Burlington, MA, USA; M4655), supplemented with Penicillin–Streptomycin (Gibco, Waltham, MA, USA; 15070063) and 10% fetal bovine serum (Scientifix, Melbourne, Australia; FBSAU-15301108). Cells were transfected using lipofectamine 2000 (Invitrogen, Waltham, MA, USA; 11668027) according to the manufacturer’s instructions. Briefly, 0.4 µL of lipofectamine 2000 was diluted in Opti-MEM (Gibco; 31985062) and added to 200 ng of diluted pGL3 plasmid DNA containing isolate LTR. An additional 4 ng of pTargetHXB2-Tat was included for Tat-transactivated conditions. Liposome–DNA complexes were incubated at room temperature for 20 min, then added onto the cells dropwise and incubated for 4–6 h before the supernatant was replaced with growth media. Cells were lysed using Luciferase Cell Culture Lysis 5X Reagent (Promega, Madison, WI, USA; E1531) 48 h post-transfection, and LTR activity was measured using a firefly luciferase assay (Promega; E4550), with luminescence measured using the CLARIOstar^®^ Plus Plate Reader (BMG LabTech, Melbourne, Australia).

Statistical analyses were performed in GraphPad Prism v10. ANOVA Kruskal–Wallis tests with Dunn’s multiple comparisons were used to compare individual LTRs to wild-type activity. Brain- versus peripheral-derived LTRs were compared using a Mann–Whitney U test. A *p*-value < 0.05 was considered statistically significant.

## Data Availability

The sequence data generated in this study have been deposited in the GenBank database under accession numbers PZ085961-PZ086026.

## Supplementary Materials

The following supporting information can be downloaded at https://www.mdpi.com/article/10.3390/ijms27073185/s1.

## Abbreviations

The following abbreviations are used in this manuscript:

A	adenine
ART	antiretroviral therapy
BR	brain
C	cytosine
C/EBP	CCAAT/enhancer binding proteins
CD4	cluster of Differentiation 4
CNS	central nervous system
CSF	cerebrospinal fluid
DNA	deoxyribonucleic acid
env	envelope
G	guanine
gDNA	genomic deoxyribonucleic acid
GI	gastrointestinal traction
HIV	human immunodeficiency virus
IQR	interquartile range
LN	lymph node
LTR	long terminal repeat
NF-κB	nuclear Factor kappa B
NNTC	National NeuroHIV Tissue Consortium
PCR	polymerase chain reaction
p-TEFb	positive transcription elongation factor b
PWH	people with HIV
RNA	ribonucleic acid
SGA	single genome amplification
Sp1	specificity protein 1
SPL	spleen
T	thymine
Tat	transactivator of transcription
TFBS	transcription factor binding site
VS	virally suppressed
