# Supplementary material for: HIV Promoters Isolated from Brain and Peripheral Tissue of Virally Suppressed PWH Are Phylogenetically and Functionally Similar
Source: Int J Mol Sci. 2026 Mar 31;27(7):3185. doi: 10.3390/ijms27073185 (PMC13073275; doi:10.3390/ijms27073185)
Supplement: Supplementary file 1 [file ijms-27-03185-s001.zip › ijms-4213820-supplementary.pdf]

Table S1. Clinical characteristics of cohort.

PWH ID		P1	P2	P3	P4	P5	Median
**Age**		62	59	64	62	52	62
**Sex**		M	F	F	M	M	–
**Ethnicity**		Not Hispanic or Latino	Hispanic or La-tino	Hispanic or La-tino	Not Hispanic or Latino	Not Hispanic or Latino	–
**Race**		Black	White	White	White	White	–
**Viral load**		UD	UD	UD	UD	UD	–
**Viral suppression (years)[a]**		3.93	2.47	2.89	7.75	6.04	3.93
**CD4+ T cells**		172	328	537	133	417	328
**ARV (at autopsy)**		DTG, TRU	RGV, TRU	ZDV, EFV, CBV, 3TC	NVP, TRU	EFV, KTA, TZV, ATR	–
**CPE score[b]**		8	7	7	8	7	7
**IPDA**	**Intact**	25	58.4	65.5	16.2	0	25
	**3′ defective**	4.2	105.9	12.5	0	24.7	12.5
	**5′ defective**	182.5	181	20.4	49.7	17.6	49.71
	**Total[c]**	211.7	345.3	98.4	65. 9	42.4	98.4

ARV: antiretroviral; ATR: atripla; CBV: combivir; CPE: CNS penetration effectiveness score; DTG: dolutegravir; EFV: efavirenz; KTA: biktarvy; NVP: nevirapine; PWH: people/person with HIV; RGV: raltegravir; TRU: truvada; UD: undetectable (<50 HIV RNA copies/mL). a missing data, b Viral suppression defined by > 2 years undetectable viral load, c not undetectable viral load for > 2 years, d CPE score ≤5 (low penetration), 6-8 (medium penetration) and ≥9 (high penetration), f CPE score for one or more ARV not defined.

Table S2. Summary of number of HIV LTRs isolated from frontal cortex brain and peripheral tissue of virally suppressed PWH.

PWH ID	Brain		Lymph Node		Gut		Spleen		All Compartments	
	**Total**	**Unique**	**Total**	**Unique**	**Total**	**Unique**	**Total**	**Unique**	**Total**	**Unique**
P1	2	2	3	3	7	7	-	-	12	12
P2	3	1	7	4	11	1	-	-	21	6
P3	5	3	9	5	-	-	-	-	14	8
P4	3	1	5	4	-	-	-	-	8	5
P5	3	3	5	5	-	-	3	2	11	10
Total	16	10	29	21	18	8	3	2	66	41

Table S3. Primer used for single genome amplification.

	Name	Direction	Sequence	HXB2 location
Round 1	Nef5′b	Forward	GGAAGCCCTCAAATATTGG	8594-8612
	CL6	Reverse	TGCTAGAGATTTTCCACAC	9701-9719
Round 2	LTR Rd 2 F	Forward	CCTGGCTAGAAGCACAA-GAGG	8963-8983
	LTR Rd 2 R	Reverse	AAGGGTCTGAGGGATCTCTAG TT	9671-9693
Round 3	LTR Rd 3 F	Forward	TGGGTTTTCCAGTCACACCTC	8993-9013
	LTR Rd 2 R	Reverse	GTCACACAACAGACGGGC	9647-9664
